# Assessment of Women’s Awareness of the Effects of Gestational Diabetes Mellitus on the Mother and Fetus in Saudi Arabia: A Cross-Sectional Study

**DOI:** 10.7759/cureus.56969

**Published:** 2024-03-26

**Authors:** Abdulrahim Gari, Sarah A Alshamlan, Muhannad Alghamdi, Manar A Ghazzawi, Mohammad A Alalawi, Elaf A Alturkustani, Renad M Alnasser

**Affiliations:** 1 Department of Obstetrics and Gynecology, Faculty of Medicine, Umm Al-Qura University, Makkah, SAU; 2 Department of Obstetrics and Gynecology, Al Salamah Hospital, Jeddah, SAU; 3 Department of Medicine, Faculty of Medicine, King Faisal University, Al-Ahsa, SAU; 4 Department of Medicine, Faculty of Medicine, Umm Al-Qura University, Makkah, SAU; 5 Department of Obstetrics and Gynecology, King Fahad Armed Forces Hospital, Jeddah, SAU; 6 Department of Medicine, Faculty of Medicine, Ibn Sina National College, Jeddah, SAU; 7 Department of Medicine, Faculty of Medicine, Al-Jouf University, Sakaka, SAU

**Keywords:** gdm, awareness, knowledge, fetus, pregnancy, gestational diabetes mellitus

## Abstract

Introduction

Gestational diabetes mellitus (GDM) is a form of glucose intolerance that arises during pregnancy, affecting a significant portion of women. It has immediate and long-term effects on both the mother and fetus, including complications like preeclampsia, premature delivery, and an increased risk of cesarean sections. A cross-sectional study among Saudi Arabia's general population, which included 979 women aged between 18 and 60, found varying levels of awareness of GDM, emphasizing the need for more research on awareness levels regarding GDM in Saudi Arabia and more educational campaigns to improve awareness.

Objectives

The study evaluates the knowledge of pregnant women about GDM and its implications for the mother and fetus. It investigates the relationship between knowledge levels and demographic factors like age, education, and socioeconomic status, aiming to identify knowledge gaps regarding this health issue and develop targeted educational initiatives.

Methodology

This was a cross-sectional study that included 979 women and was conducted using a Google Forms (Google Inc., Mountainview, CA) questionnaire. The questionnaire covered demographics and explored the knowledge level of women about the impact of GDM on the mother and fetus. Statistical analysis was implemented by IBM SPSS software version 27.0 (IBM Corp., Armonk, NY), with a 5% significance level. Ethical approval was sought, emphasizing anonymous data collection. We did not collect any identifying or private information from participants, and all responses were kept confidential.

Results

A study of 979 women revealed that their knowledge of GDM was significantly influenced by their age, gestational age, and the number of prior deliveries (p-value < 0.05). The total mean knowledge score for women's correct responses stood at 7.62 (±4.49). The study found that a majority of women, exceeding 60%, accurately answered certain questions about GDM, such as its association with heightened risks, neonatal intensive care unit (NICU) admissions, cesarean section likelihood, high birth weight, and preeclampsia. However, less than 30% could answer yes to questions that indicated that GDM could increase the risk of shoulder dystocia, hypoglycemia at birth, premature rupture of membranes, postpartum hemorrhage, and vacuum delivery.

Conclusion

There is a need for targeted educational initiatives, particularly focusing on knowledge gaps that women are lacking regarding GDM. Age and prior deliveries were identified as significant determinants of knowledge levels.

## Introduction

Gestational diabetes mellitus (GDM) is described as any degree of glucose intolerance with onset or first acknowledgment occurring amid pregnancy (second or third trimester) [1]. Gestational diabetes mellitus poses a significant public health concern, impacting a considerable portion of the female population [2]. This condition brings about immediate and prolonged effects on the mother and the developing fetus [2]. Gestational diabetes mellitus is linked to a range of conditions; these encompass an elevated occurrence of preeclampsia and unfavorable childbirth outcomes like premature delivery, an elevated risk of C-sections, and birth lacerations or trauma. Additional aspects comprise the potential for fetal demise during the third trimester, low blood sugar and calcium levels in newborns, a high red blood cell (RBC) count, and respiratory distress syndrome (RDS) [3].

In 2019, the International Diabetes Foundation provided an estimation indicating that around 16% of women who gave birth experienced hyperglycemia in pregnancy to some extent, with approximately 85.1% of these cases attributed to GDM [4]. Various kinds of research have been undertaken worldwide to assess the levels of knowledge and awareness about GDM. Multiple assessments have been carried out in different regions of Saudi Arabia to promote GDM awareness among pregnant women for a healthy mother and baby outcome [1]. A study done in Jizan involved 440 pregnant women chosen via a simple random sampling method. The outcome showed that the prevalence of GDM among expectant women was 8.2% [4]. Another study done in 2018 reported that Saudi Arabia is the third-highest country with a prevalence of GDM (22.9%) [5].

In our research, we want to evaluate the overall level of knowledge among pregnant women regarding GDM and its impact on both mother and fetus and assess the association between the levels of knowledge about GDM and certain demographic factors such as age, education, and socioeconomic status.

## Materials and methods

Methods

A cross-sectional online survey was carried out in Saudi Arabia, between August 2023 and October 2023. The study was aimed at Saudi women who lived in Saudi Arabia and were between 18 and 60 years of age. Data collection was carried out by means of a self-administered questionnaire. We used a simple random sampling method. Before filling out the questionnaire, all participants were provided with an informed consent form.

Data collection tool and procedure

The authors developed the questionnaire in response to a thorough review of relevant literature. In order to ensure clarity and simplicity, the questionnaire was reviewed by experts in the field of obstetrics and gynecology, and all items were considered relevant for the purpose of the study. In order to make it easier for the population to read and understand, the survey was been designed in Arabic. The questionnaire used contained five sections that involved questions related to the participants' sociodemographic data, the effects of GDM on mothers, and neonatal outcomes. Regarding the effects of GDM on mothers, there were eight questions with the options of "yes", "no", and "I don’t know" to respond to common maternal complications due to GDM. Concerning awareness of the effects of GDM on neonatal outcomes, to measure the level of awareness, participants answered 10 yes-or-no questions about the effects of GDM on neonatal outcomes. The first page of the questionnaire was designated for informed consent. An electronic Google Forms (Google Inc., Mountainview, CA) survey was used and distributed on different social media platforms, such as WhatsApp (Meta Platforms, Inc., Menlo Park, CA), X, formerly known as Twitter (X Corp., San Francisco, CA), and Telegram (Telegram FZ LLC, Dubai, UAE). Using features of Google Forms such as “required to proceed” to make sure the study criteria would be fulfilled, the following question was provided at the beginning of the questionnaire: "Are you currently pregnant?" In case the reply was "yes," the participant would proceed to go through other questions within the survey. In case the reply was "no," the participants would go through other questions measuring their awareness level via the questionnaire form. A combined system of codes, numbers, and pseudonyms was set up to ensure the confidentiality of participants' information. Only researchers had access to the data.

Sample size

The sample size was calculated using the Raosoft online sample calculator (Raosoft Inc., Seattle, WA) based on the population size in the Saudi Arabian region (approximately 37,045,512). With a 95% confidence interval and a 5% margin of error, the required number of participants was at least 385 for the study. However, we recruited a higher number of participants due to the possibility of data loss. Additionally, we used a simple random sampling method.

Ethical considerations and confidentiality

The biomedical ethics committee of the University of Umm Al-Qura, Makkah, Saudi Arabia, approved this research (approval number: HAPO-02-K-012-2023-09-1763). Survey responses were collected anonymously. We did not collect any identifying or private information from the participants and all responses were kept confidential.

Statistical analysis

The research involved an extensive statistical analysis of the collected data, utilizing descriptive and inferential methodologies. Analysis of participants' sociodemographic characteristics included calculating frequencies and percentages for categorical variables, as well as means, standard deviations, medians, and interquartile ranges (IQRs) for continuous variables. Using a set of 19 knowledge-based questions, incorrect answers were assigned zero values, while correct ones were given a value of one, allowing for the calculation of total knowledge scores per participant. To assess variations in mean knowledge scores across different sociodemographic factors, the Mann-Whitney U test and the Kruskal-Wallis test were employed. Statistical significance was determined at a p-value of 0.05 or lower with a 95% confidence interval. All statistical analyses were performed using IBM SPSS Statistics for Windows, version 27.0 (IBM Corp., Armonk, NY).

## Results

A total of 979 women voluntarily consented to participate (Table 1). Among these participants, 385 individuals (39.3%) fell within the age range of 25 to 35 years old, while 427 women (43.6%) were older than 35 years. The majority (915 individuals, 93.5%) were of Saudi nationality, and a significant proportion held at least a university degree, totaling 498 individuals (56.7%). On average, the participants had a mean height of 158.02 (±6.88) centimeters and a mean weight of 66.74 (±16.77) kilograms. Regarding their obstetric history, a minority were pregnant during the study (108 individuals, 11.0%), while 106 participants (10.8%) had one baby, and 91 individuals (9.3%) delivered at a gestational age of less than 37 weeks (preterm). Most women were multiparous (608 individuals, 62.1%).

**Table 1:** Sociodemographic characteristics of the participants n: number; %: percentage; SD: standard deviation

Question	Answers	Frequency n (%)
Age (years)	<25	167 (17.1)
	25-35	385 (39.3)
	>35	427 (43.6)
Nationality	Saudi	915 (93.5)
	Non-Saudi	63 (6.5)
Educational status	Unschooled	14 (1.6)
	Secondary school or less	247 (28.1)
	Bachelor’s degree	498 (56.7)
	Postgraduate (Master of Science (MSc), PhD)	119 (13.6)
Are you currently pregnant?	Yes	108 (11.0)
	No	871 (89.0)
Number of pregnancies	One	106 (10.8)
	Two or more	2 (0.2)
Gestational age (weeks)	<37	91 (9.3)
	37-40	16 (1.6)
	>40	1 (0.1)
Parity	Nulliparous	247 (25.1)
	Para 1	124 (12.7)
	Multiparous	608 (62.1)
Height (cm) mean (±SD)	158.02 (±6.88)	
Weight (kg) mean (±SD)	166.74 (±16.77)	

Regarding women's overall knowledge concerning GDM, out of a total score of 19, the total mean knowledge score derived from women's responses stood at 7.62 (±4.49). A deeper analysis revealed that a substantial majority, exceeding 60%, accurately responded to five specific questions regarding GDM. These included understanding its association with heightened risks such as preterm delivery (n = 687, 70.2%), neonatal intensive care unit (NICU) admissions exceeding 24 hours (n = 660, 67.4%), increased cesarean section likelihood (n = 640, 65.4%), increased odds of high birth weight (n = 633, 64.7%), and its correlation with a greater risk of preeclampsia (n = 593, 60.6%). Conversely, the correct answer rates were notably lower; below 30% for six crucial questions which were GDM's potential connection with risks like breech delivery (n = 169, 17.3%), premature rupture of membranes (n = 224, 22.9%), vacuum delivery (n = 247, 25.2%), hypoglycemia at birth (n = 267, 27.3%), postpartum hemorrhage (n = 274, 28.0%), and shoulder dystocia (n = 290, 29.6%). Meanwhile, the correct answer rates for the remaining eight questions fell within the range of 29.6% to 60.6%, indicating varying levels of awareness among participants regarding their knowledge of GDM (Table 2).

**Table 2:** Assessment of the overall level of knowledge among women regarding GDM n: number; %: percentage; SD: standard deviation; IQR: interquartile range; DM: diabetes mellitus; GDM: gestational diabetes mellitus; CS: cesarean section; PPH: postpartum hemorrhage; NICU: neonatal intensive care unit; RDS: respiratory distress syndrome

Question	Correct answer	Frequency n (%)
Knowledge score (total of 19)	Mean (±SD)	7.62 (±4.49)
	Median (IQR)	7 (4-11)
Gestational DM screening should be done at (weeks)	24-27	416 (42.5)
Do you think GDM increases the risk of vacuum delivery?	Yes	247 (25.2)
Do you think GDM increases the risk of CS?	Yes	640 (65.4)
Do you think GDM increases the risk of polyhydramnios?	Yes	321 (32.8)
Do you think GDM increases the risk of PPH?	Yes	274 (28.0)
Do you think GDM increases the risk of rupture of membranes?	Yes	224 (22.9)
Do you think GDM increases the risk of placental abruption?	Yes	322 (32.9)
Do you think GDM increases the risk of preeclampsia?	Yes	593 (60.6)
Do you think GDM increases the risk of preterm delivery?	Yes	687 (70.2)
Do you think GDM increases the risk of high birth weight?	Yes	633 (64.7)
Do you think GDM increases the risk of NICU admissions >24 hours?	Yes	660 (67.4)
Do you think GDM increases the risk of RDS?	Yes	313 (32.0)
Do you think that GDM increases the risk of hypoglycemia at birth?	Yes	267 (27.3)
Do you think that GDM increases the risk of hyperbilirubinemia?	Yes	354 (36.2)
Do you think GDM increases the risk of shoulder dystocia?	Yes	290 (29.6)
Do you think GDM increases the risk of breech delivery?	Yes	169 (17.3)
Do you think GDM increases the risk of stillbirth?	Yes	407 (41.6)
Do you think GDM increases the risk of neonatal death?	Yes	344 (35.1)
Do you think GDM increases the risk of congenital neonatal anomalies?	Yes	295 (30.1)

As described in Table 3, there was a variation in mean scores of GDM knowledge among women based on their sociodemographic characteristics. Age emerged as a significant determinant of women's GDM knowledge level (p-value < 0.001). Notably, women below the age of 25 exhibited higher awareness of the disease, with a mean score of 9.01 compared to higher age groups, who recorded mean scores of 7.62 and 7.07, respectively. Gestational age at delivery also proved influential in shaping knowledge of GDM (p-value < 0.001). Women who delivered at 40 weeks of gestational age demonstrated the highest mean score of 7.87, compared to counterparts with deliveries below 37 weeks (preterm) or between 37 and 40 weeks, who scored mean values of 5.76 and 5.00, respectively. The number of prior deliveries also impacted women's GDM knowledge (p-value = 0.006). Surprisingly, women without prior deliveries exhibited the highest knowledge level of the disease, scoring a mean of 8.28, in contrast to those with one or more deliveries, whose mean scores were 6.69 and 7.54, respectively. However, sociodemographic factors such as nationality, education status, and conception status did not significantly influence women's levels of GDM knowledge (p-value > 0.05).

**Table 3:** Assessment of knowledge level scores among women regarding GDM based on sociodemographic data ^a^Mann-Whitney U Test; ^b^Kruskal-Wallis Test; SD: standard deviation

Variable		Mean (±SD)	p-value
Age (years)^b^	<25	9.01 (±5.05)	0.001
	25-35	7.62 (±4.27)	
	>35	7.07 (±4.35)	
Nationality^a^	Saudi	7.58 (±4.51)	0.496
	Non-Saudi	8.20 (±4.29)	
Educational status^b^	Unschooled	5.00 (±5.94)	0.435
	Secondary school or less	7.39 (±4.36)	
	Bachelor’s degree	7.70 (±4.49)	
	Postgraduate (Master of Science (MSc), PhD)	8.06 (±5.14)	
Are you currently pregnant?^a^	Yes	5.59 (±3.73)	0.189
	No	7.87 (±4.52)	
Number of pregnancies?^a^	One	7.87 (±4.52)	0.189
	Two or more	7.50 (±6.36)	
Gestational age (weeks)^b^	<37	5.76 (±3.72)	0.001
	37-40	5.00 (±3.68)	
	>40	7.87 (±4.52)	
Parity^b^	Nulliparous	8.28 (±5.25)	0.006
	Para 1	6.69 (±4.02)	
	Multiparous	7.54 (±4.21)	

## Discussion

The study aimed to assess Saudi women's knowledge of GDM and its impact on maternal and fetal health. Furthermore, the study looked into potential sociodemographic factors that could affect their level of awareness.

In our study, women's overall knowledge of GDM was 7.62 (±4.49). A study in India by Thomas et al. (2020) reported that only 33 (6.3%) were aware of GDM, but 490 (93.69%) individuals had no idea what the illness was [6]. However, prior research conducted in a number of locations outside of India revealed that the majority of pregnant women are typically unaware of GDM [7]. In another study, it was found that the mean percent mark of the multitude of women's awareness regarding GDM as far as anyone is concerned was nearly 46.1%. There is a lack of awareness about GDM among pregnant women, especially in rural areas [8]. The findings of our study are in line with those of an Indian study, where 25.8% of the participants were not well educated, 56.7% had average knowledge, and 17.5% had good knowledge about GDM. [9].

In this study, the correct knowledge about GDM screening at weeks 24-27 was in 416 patients (42.5%). To compare this finding to a study done by Bhavadharini et al. (2017) in Tamil Nadu, it was found that 88.7% of the women from the urban area knew that it was important to screen for GDM. However, the majority of women in the urban area (64.4%) felt that screening should be carried out during the first trimester itself, while people from rural areas were less aware of when they should undergo screening [10]. In our study, more than half of the participants knew the increased risk of cesarean section likelihood among GDM patients (n = 640, 65.4%). Many studies were consistent with this point. Gorgal et al. (2012) found that women with GDM had a non-elective cesarean section rate of 19.5%, while women without the disease had a rate of 13.5%. For women with GDM, the crude relative risk of a cesarean section was 1.45 (95% CI 1.04-2.02). The correlation between GDM and non-elective cesarean sections was still positive and statistically significant (relative risk (RR) = 1.52; 95% CI 1.06-2.16), even when covariates were taken into account. It was discovered that GDM represented a risk for non-elective cesarean procedures [11].

Sixty percent accurately answered five specific GDM-related questions. These included understanding its association with heightened risks such as preterm delivery, NICU admissions exceeding 24 hours, increased cesarean section likelihood, increased odds of high birth weight, and a greater risk of preeclampsia. Increased awareness of these conditions could improve maternal and neonatal outcomes.

On the contrary, the correct answer rates were significantly lower, falling below 30% for six critical questions which were GDM's potential association with risks such as breech delivery, membrane rupture, vacuum delivery, hypoglycemia at birth, postpartum hemorrhage, and shoulder dystocia. This finding may indicate the need to raise awareness about the conditions mentioned in these questions and their relationship to GDM.

In our study, a significant portion (n = 593, or 60.6%) correctly knew that GDM increased the risk of preeclampsia. We compared our findings to a study in Ghana, where the prevalence of inadequate knowledge of preeclampsia was 88.6% (mean score = 55.5 ± 4.3), while the prevalence of adequate knowledge of preeclampsia was only 11.4% (mean score = 76.3 ± 5.9) [12]. In Malaysia, a study by Teng and Keng (2016) found only 18.4% of women have adequate knowledge of preeclampsia [13]. Other studies by Savage and Hoho (2016) [14] and Eze et al. (2018) [15] reported that 59% and 60% of Tanzanian women had inadequate knowledge of preeclampsia, respectively.

In our research, participants accurately responded to specific questions about the effects of GDM on both mothers and fetuses. For example, 32.9% answered that GDM increases the risk of placental abruption, and 64.7% recognized its association with high birth weights.

A study conducted in Saudi Arabia highlighted that 71.1% of participants were uncertain about whether GDM could elevate the risk of placental abruption, indicating insufficient knowledge among pregnant women regarding GDM's effects on mothers and neonates [1].

Additionally, another study discovered a significant difference in awareness levels regarding GDM between women diagnosed with GDM (the treatment group) and those without GDM (the control group). The mean awareness score was notably higher in the treatment group (mean ± SD = 2.6 ±1.8) than in the control group (mean ± SD = 2.14 ±1.8) with a p-value of 0.012 [5].

In our study, 29.6% accurately replied that GDM increases the risk of shoulder dystocia, and 17.3% correctly reported that it also increases the risk of breech delivery. Another Saudi study demonstrated that 70.7% and 68.5% of the participants were unsure of whether GDM could increase the risk of shoulder dystocia and breech delivery, respectively. Regarding the fact that GDM increases the risk of postpartum hemorrhage, 28.0% of our participants correctly answered it, whereas in another Saudi study by Salhi et al. (2019), 65.7% of the participants were unsure of whether GDM can increase the risk of postpartum hemorrhage or not, which is higher than the percentage we found [1]. Age emerged as a significant determinant of women's GDM knowledge level in our study (p-value < 0.001). Notably, women below the age of 25 exhibited higher awareness of the disease, with a mean score of 9.01 compared to higher age groups, who recorded mean scores of 7.62 and 7.07, respectively. We compared our findings to a study in which the age variable had the highest predictive power for awareness (Exp (β) = 1.36, Wald = 32.2, p <.01). After adjusting for all other model parameters, higher-age group respondents were 1.36 times more likely to report awareness than younger respondents. [6]. Women over 35 years of age were more accurate in answering questions on GDM compared to women under 25 years old, according to the study by Gastrich et al. (213) [16].

The number of prior deliveries in our study also impacted women's GDM knowledge (p-value = 0.006). Women without prior deliveries exhibited the highest knowledge level of the disease, scoring a mean of 8.28, in contrast to those with one or more deliveries, whose mean scores were 6.69 and 7.54, respectively. The number of pregnancies was also a predictor of awareness of GDM. The respondents with the highest number of pregnancies were 0.35 times more aware of GDM when compared with those who had fewer gestations [6]. The experience of multiple pregnancies could serve as a resource for better information on health. However, these findings were not consistent with other studies reported by Carolan et al. (2010) and Shriraam et al. (2013), which stated that age and the number of pregnancies did not have any influence on awareness of GDM [7,9]. Our results may simply be because of selection bias or sample size. We found that knowledge level scores regarding GDM based on their education level were not significantly different, with a p-value of 0.435. To compare our findings to a study conducted in Bangladesh in which university-educated students (university-educated vs. college vs. secondary vs. primary vs. unschooled; 3.53 vs. 2.74 vs. 2.35 vs. 1.90 vs. 0.75, p < 0.001). It was found that illiterate participants had the lowest knowledge score, whereas participants who had a university education showed the highest knowledge score [17].

Limitations

There are limitations to this study, yet it is informative. Sample bias could be introduced by its web-based cross-sectional design, which favors individuals with internet access. Recall or social desirability bias could affect the results of online surveys that rely on self-reporting. The results may only apply to the sample population, and the reliability of the questionnaire could affect how accurate the responses are. Establishing causation or tracking changes over time is made more difficult by the cross-sectional nature of the study. Qualitative approaches, a diversified sample to increase generalizability, and longitudinal studies should be considered for future research.

## Conclusions

This study shed light on women's knowledge about GDM in Saudi Arabia and its related implications. The results emphasize the necessity for targeted educational programs, particularly those focusing on lesser-known aspects of GDM. Although most participants displayed awareness of certain GDM risks, significant gaps in understanding crucial aspects were evident. The low knowledge levels highlight the need for tailored educational interventions for specific demographic groups. This research emphasizes the ongoing necessity to enhance public understanding and support maternal and neonatal health. Collaborative efforts involving healthcare providers and community organizations are crucial in launching awareness campaigns targeting women of childbearing age to improve maternal and newborn health outcomes.

## References

[1] Salhi AA, Alshahrani MS, Alyamin MM (2019). Assessment of the knowledge of pregnant women regarding the effects of GDM on mothers and neonates at a maternal and children hospital in Najran, Saudi Arabia. Int J Med Dev Ctries.

[2] Alnaim A (2020). Knowledge of gestational diabetes mellitus among prenatal women attending a public health center in Al-Khobar, Saudi Arabia. Egypt J Hosp Med.

[3] Bener A, Saleh NM, Al-Hamaq A (2011). Prevalence of gestational diabetes and associated maternal and neonatal complications in a fast-developing community: global comparisons. Int J Womens Health.

[4] Alnaeem LS (2019). Awareness of gestational diabetes among antenatal women at the King Fahd Military Medical complex hospital in Dhahran, Saudi Arabia. Egypt J Hosp Med.

[5] Quaresima P, Visconti F, Interlandi F (2021). Awareness of gestational diabetes mellitus foetal-maternal risks: an Italian cohort study on pregnant women. BMC Pregnancy Childbirth.

[6] Thomas S, Pienyu E, Rajan SK (2020). Awareness and knowledge about gestational diabetes mellitus among antenatal women. Psych Community Health.

[7] Carolan M, Gill GK, Steele C (2012). Women's experiences of factors that facilitate or inhibit gestational diabetes self-management. BMC Pregnancy Childbirth.

[8] Deepa M, Bhansali A, Anjana RM (2014). Knowledge and awareness of diabetes in urban and rural India: The Indian Council of Medical Research India Diabetes Study (Phase I): Indian Council of Medical Research India Diabetes 4. Indian J Endocrinol Metab.

[9] Shriraam V, Rani MA, Sathiyasekaran BW, Mahadevan S (2013). Awareness of gestational diabetes mellitus among antenatal women in a primary health center in South India. Indian J Endocrinol Metab.

[10] Bhavadharini B, Deepa M, Nallaperumal S, Mohan AR, Viswanathan M (2017). Knowledge about gestational diabetes mellitus amongst pregnant women in South Tamil Nadu. J Diabetes.

[11] Gorgal R, Gonçalves E, Barros M, Namora G, Magalhães A, Rodrigues T, Montenegro N (2012). Gestational diabetes mellitus: a risk factor for non-elective cesarean section. J Obstet Gynaecol Res.

[12] Fondjo LA, Boamah VE, Fierti A, Gyesi D, Owiredu EW (2019). Knowledge of preeclampsia and its associated factors among pregnant women: a possible link to reduce related adverse outcomes. BMC Pregnancy Childbirth.

[13] Teng Teng, S.P. and S.L. Keng (2016). Knowledge of preeclampsia among antenatal women in a tertiary referral teaching hospital. The. Malays J Nursing.

[14] Savage AR, Hoho L (2016). Knowledge of pre-eclampsia in women living in Makole Ward, Dodoma, Tanzania. Afr Health Sci.

[15] Eze ED, Barasa A, Adams MD, Rabiu KM, Ezekiel I, Sulaiman SO, Ponsiano N (2018). Determination, knowledge and prevalence of pregnancy-induced hypertension/eclampsia among women of childbearing age at Same District Hospital in Tanzania. Int J Med Med Sci.

[16] Gastrich MD, Peck S, Janevic T, Bachmann G, Lotwala N, Siyam A (2013). Gestational diabetes mellitus: an educational opportunity. J Diabetes Nurs.

[17] Bhowmik B, Afsana F, Ahmed T (2018). Evaluation of knowledge regarding gestational diabetes mellitus: a Bangladeshi study. Public Health.

